# Direct Visualization of Actin Filaments and Actin-Binding Proteins in Neuronal Cells

**DOI:** 10.3389/fcell.2020.588556

**Published:** 2020-11-26

**Authors:** Minkyo Jung, Doory Kim, Ji Young Mun

**Affiliations:** ^1^Neural Circuit Research Group, Korea Brain Research Institute, Daegu, South Korea; ^2^Department of Chemistry, Research Institute for Convergence of Basic Sciences, Institute of Nano Science and Technology, Research Institute for Natural Sciences, Hanyang University, Seoul, South Korea

**Keywords:** electron microscopy, super-resolution microscopy, correlative light and electron microscopy, actin binding protein, actin, neuronal cell

## Abstract

Actin networks and actin-binding proteins (ABPs) are most abundant in the cytoskeleton of neurons. The function of ABPs in neurons is nucleation of actin polymerization, polymerization or depolymerization regulation, bundling of actin through crosslinking or stabilization, cargo movement along actin filaments, and anchoring of actin to other cellular components. In axons, ABP–actin interaction forms a dynamic, deep actin network, which regulates axon extension, guidance, axon branches, and synaptic structures. In dendrites, actin and ABPs are related to filopodia attenuation, spine formation, and synapse plasticity. ABP phosphorylation or mutation changes ABP–actin binding, which regulates axon or dendritic plasticity. In addition, hyperactive ABPs might also be expressed as aggregates of abnormal proteins in neurodegeneration. Those changes cause many neurological disorders. Here, we will review direct visualization of ABP and actin using various electron microscopy (EM) techniques, super resolution microscopy (SRM), and correlative light and electron microscopy (CLEM) with discussion of important ABPs in neuron.

## Introduction

**FIGURE 1:**
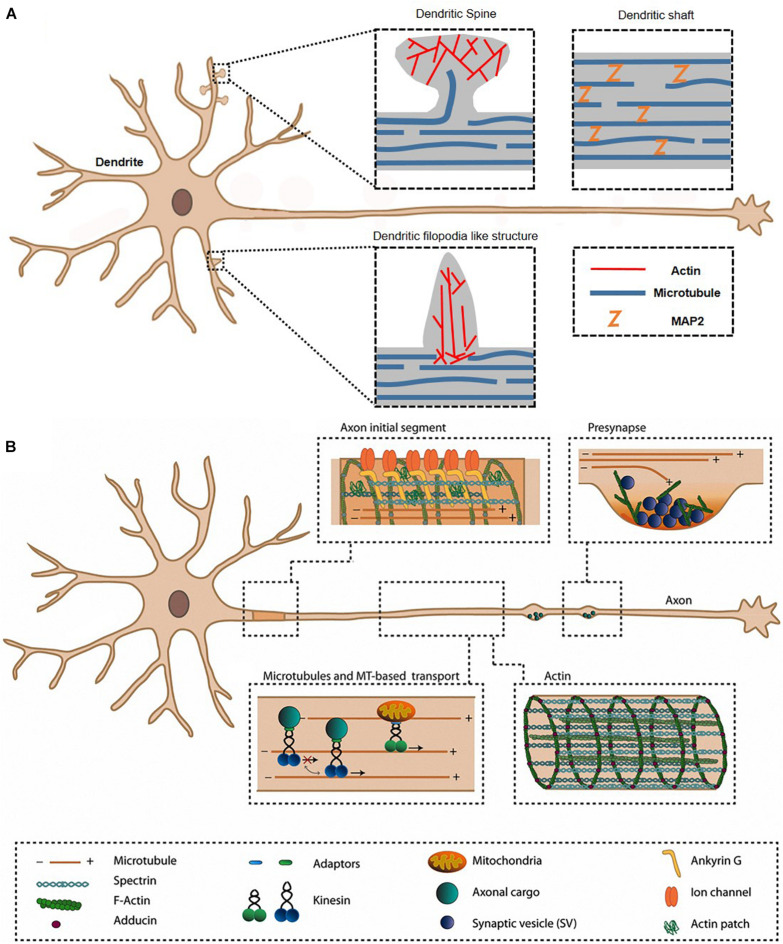
Axon and dendrite in a neuron. The morphological characteristics are mainly related to actin, ABPs, and other cytoskeletons in dendrite **(A)** and axon **(B)**. ABP, actin-binding protein. Reprinted by permission from Kevenaar and Hoogenraad (2015).

**FIGURE 2:**
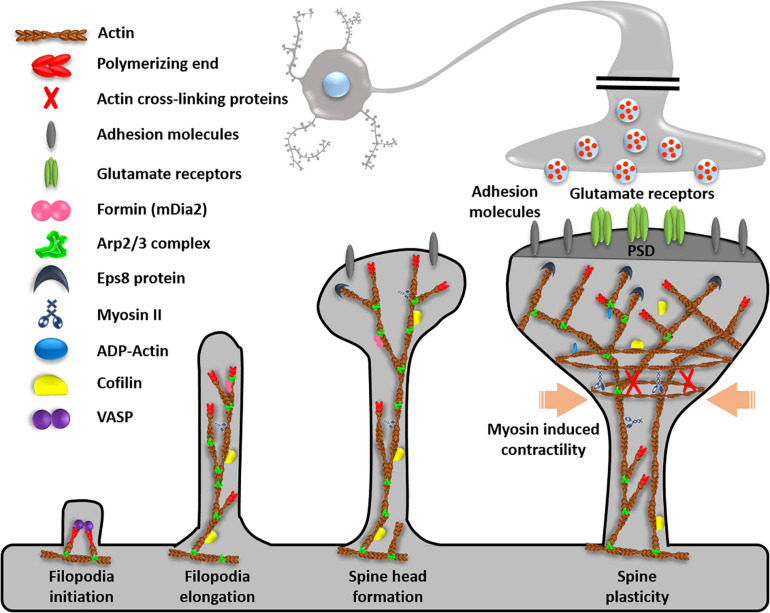
Function of ABPs in dendrites. Dendrites contain ABPs for filopodia initiation, elongation, spine head formation, and spine plasticity. Reprinted and modified by permission from Elsevier (Soria Fregozo and Perez Vega, 2012).

Neurons are specialized cells with long processes or connections in the nervous system. Each neuron has two types of cytoplasmic protrusions from the neuronal cell body, an axon and a dendrite (Figure 1). The length of an axon is the distance between one neuron and a target neuron, which varies from tens of micrometers to 1 m. The function of axon for transmission of information to different neurons is essential for brain function, therefore axons normally form a connection with the correct target and maintain their structure. Because each neuron has a single axon, axon dysfunction is both the result and the main cause of many neurological disorders, such as loss of cognitive ability, general paralysis, paraplegia, and loss of sensory function. Another protrusion from neuron is dendrites, and the main function is receiving signals from other neurons. In dendrite, spines receiving signals can be classified into mature spines, mushroom-shaped and immature spines (filopodia), or thin spines with a hairpin-like fine head <3 μm in length (Figure 2). Actin filaments are a part of the cytoskeleton and form the specific axon and dendrite morphology (Dogterom and Koenderink, 2019). In developing neurons, dynamic regulation of actin polymerization and organization mediates axon morphogenesis and path finding to synaptic targets. Changes in the axon shape such as branching, branch retraction, axonal arbor morphology depend on actin filament dynamics. Actin filaments do not function in a naked state, and actin binding proteins (ABP) s regulate all aspects of actin, that is, actin filament dynamics and organization are regulated by various ABPs. In mature axons, stable F-actin and ABPs play a scaffolding role and maintain axon integrity through actin ring and help transport organelles. In addition, F-actin and ABPs mediate vesicular trafficking and regulate neurotransmitter release in mature axon terminals. Actin remodeling through ABPs in synaptic boutons also plays a crucial role in postsynaptic terminal plasticity. In postsynaptic terminal, the dendritic spine length, branching, spine density, shape of spine, and spine distribution (actin-rich protrusions from the dendritic shaft), and motility respond morphologically to various physiological stimuli depending on actin and ABPs. Dysfunction of synapse plasticity causes neurological and psychiatric disorder, which includes Alzheimer’s disease (AD), Parkinson’s disease (PD), Huntington’s disease (HD), schizophrenia, autism, and depression (Ma et al., 2012; Borovac et al., 2018). Therefore, researchers are trying to understand the mechanism of interaction of actin and ABPs in axon and dendrite in order to find ways to prevent neuronal disorders.

As ABPs, the Arp2/3 complex binds with F-actin and nucleates a new filament from an existing actin filament. Arp2/3 complex deficiency induces excessive growth, exclusive axon branching, and loss of dendritic spine maturation and enlargement (Strasser et al., 2004; Pinyol et al., 2007). Formins are unbranched actin nucleators (Kuhn and Geyer, 2014), and involved in proper axon development (Matusek et al., 2008) through deep actin network formation. A high formin amount causes long F-actin bundles to push out filopodia and promotes long actin filament polymerization (Hotulainen et al., 2009). Filament-severing proteins, such as actin-depolymerizing factor (ADF) and cofilin-1 accelerated actin turnover (Sarmiere and Bamburg, 2004), because cofilin is a major actin depolymerization factor for regulating actin length (Cichon et al., 2012). Cofilin-1 activity is necessary for neurite growth and growth cone turning, receptor trafficking, dendritic spinogenesis, and dynamic synaptic plasticity (Noguchi et al., 2016). As a linker to form actin networks, there are reports about ABPs tropomodulin (Gray et al., 2017), filamin (Cho et al., 2015), neurofascin (Winkelman et al., 2014), spectrin (Unsain et al., 2018), adducin (Leite et al., 2016), and ERM proteins (ezrin, radixin, moesin) (Marsick et al., 2012). Among them, we will show details of main proteins such as Arp2/3, spectrin, neurofascin, and adducin through direct visualization techniques.

Electron Microscopy (EM) is one of the primary methods of choice for visualizing actin and ABP, as it is capable of achieving a nanometer resolution. However, the application of conventional EM for live or wet biological samples is limited due to the need for high-vacuum conditions (Kim et al., 2015). Moreover, the molecular specificity is limited in EM imaging as electron-dense heavy metals used for increasing the electron contrast of biological samples often stain lipids or proteins non-specifically. Although several molecular structures can be identified by their characteristic shapes from advanced EM images, such identifiable molecular structures are relatively few, and the characteristic structures of macromolecules are not known in most cases (Kim et al., 2015). Immuno-EM using antibodies conjugated with gold nanoparticles can be used to localize specific targets in EM, but it suffers from a low labeling efficiency and limited multi-target imaging (Griffiths and Hoppeler, 1986; Robinson et al., 2000). These limitations can be overcome using advanced EM techniques such as cryo-techniques, light microscopy (LM) with super resolution, and correlative light and electron microscopy (CLEM). Although LM exhibits diffraction-limited spatial resolution (∼300 nm), it enables the simultaneous visualization of multiple targets with high molecular specificity. Live and wet biological samples can also be imaged using light microscopy. The recent development of super-resolution fluorescence microscopy (SRM) has enabled substantially increased spatial resolution of light microscopy (∼10 nm) by overcoming the diffraction limit (Hell and Wichmann, 1994; Betzig et al., 2006; Rust et al., 2006; Huang et al., 2009).

In this review, we focused on the application of (1) EM such as single particle analysis, electron tomography, unroofing and, etching with cryo-techniques, (2) SRM such as STORM, PALM, and STED, (3) CLEM to the study of the molecular architecture of actin and ABP, since these are the most widely used techniques for such molecular ultrastructural studies.

## Visualization Techniques in the Nanoscale to Study Actin and Actin-Binding Proteins

### Electron Microscopy

Electron microscopy is good tool for study actin and ABPs because of its nano scale resolution. Traditionally, actin and actin ABPs were observed by conventional EM such as negative stained EM and etching techniques. However, they have some limitations as we mentioned in introduction. Recently, advanced techniques including cryo-TEM, cryo-electron tomography, and CLEM have been used for the study. We will discuss three methods including single particle analysis, electron tomography with freeze-etching and unroof, and immuno gold staining among various EM techniques with their findings. As a single particle analysis with negative stained EM or cryo-EM showed proteins’ interaction in angstrom resolution level. It can reveal of ABPs’ binding mechanism to actin and their competition. Electron tomography with unroof and etching showed intervals the protein’s interaction to actin with higher resolution. Immuno-gold labeling showed specific ABP’s detailed locations in cells.

#### Single Particle Analysis Using Negative-Stained EM and Cryo-EM

**FIGURE 3:**
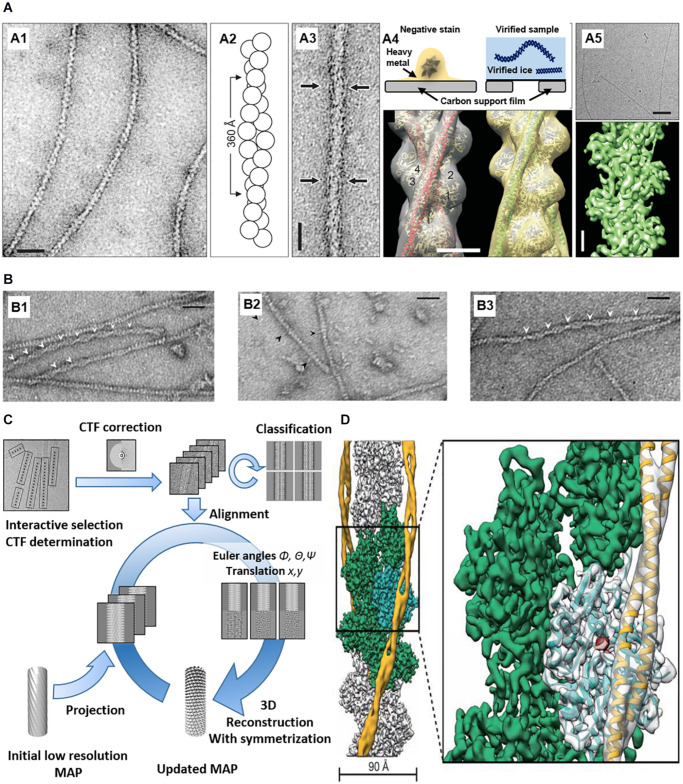
EM imaging and 3D reconstruction of actin with ABPs *in vitro*. **(A) (A1)** Negatively stained F-actin. **(A2)** F-actin model based on EM. **(A3)** Negatively stained actin and ABP (arrows). **(A4)** (Top) Comparison between negative-stained EM and cryo-EM. (Bottom) 3D reconstruction of actin and ABPs based on negative staining data. **(A5)** Cryo-EM of F-actin (top) and 3D reconstruction at ∼5.5 Å resolution (bottom) Reprinted by permission from Korean Society of Microscopy (Craig, 2017). Scale bars = 200 Å **(A1,A3)**, 50 Å **(A4)**, 500 Å (**(A5)**, top), 20 Å (**(A5)**, bottom). **(B) (B1)** Actin filaments bound with cofilin-rod. Arrowheads show the intersection of the cofilin-rod clusters. **(B2)** Cofilin-rod molecules are rarely bound to actin filaments and identified by a rod-shaped structure (black arrowheads). **(B3)** Actin filaments with bound cofilin molecules (without rod fusion). Scale bars = 25 nm. Reprinted by permission from Ngo et al. (2015). **(C)** 3D reconstruction workflow. Reprinted by permission from Elsevier (Fromm and Sachse, 2016). **(D)** 3D reconstruction of actin with ABP (tropomyosin) from cryo-EM. PDB fitted to 3D EM volume. Scale bars = 90 Å. Reprinted by permission from Springer Nature (Von Der Ecken et al., 2015).

Simple and reliable EM technique combined by image processing and 3D reconstruction (Figure 3) can be used to visualize ABPs. EM and 3D reconstruction of purified ABPs and actin show ABP-binding sites for actin filaments *in vitro* (Narita and Maeda, 2007; Egelman, 2010; Behrmann et al., 2012) are used. Negative-stained EM of F-actin itself and F-actin with ABPs has been conventionally used for structural studies of actin with specific ABPs. Negative staining is a simple sample preparation method in which protein samples are embedded in a thin layer of dried heavy metal salt to increase specimen contrast. After EM imaging in low-dose mode, the straightened single filaments from the images are used for helical reconstruction or iterative helical real space reconstruction (IHRSR). Averaging the reconstructions shows the 3D structure density, and the known X-ray crystal structure of each protein can be fitted to the 3D structure. The technique shows the binding sites of cortactin (Pant et al., 2006), ADF/cofilin (Galkin et al., 2011), vinculin (Thompson et al., 2014), drebrin (Grintsevich et al., 2010) to actin.

Cryo-EM is applied to reveal further details because of its high resolution without staining and dehydration. Compared to negative-stained EM with ∼20 Å resolution, cryo-EM can reach 1–2 Å resolution (Bartesaghi et al., 2015; Merk et al., 2020). For decades, structural biologists have conventionally used X-ray crystallography involving crystallizing proteins. X-ray crystallography gives high-quality images of structures, but it is difficult to make crystals. Cryo-EM does not use crystallized proteins, and uses vitrified pure water including a protein solution in a hydrophilic carbon film, which is plunged into a cryogen, such as liquid ethane. Frozen, hydrated protein samples are embedded in a thin layer of vitreous ice, which is imaged by cryo-EM using direct electron detection with complementary metal oxide semiconductor (CMOS)-based sensors or a charge-coupled device (CCD). The first cryo-EM of actin was performed by Trinick et al. (1986) at 40 Å resolution, and recent data of phalloidin-bound F-actin was reconstructed at 3.3 Å resolution (Das et al., 2020). Using this technique, Galkin et al. (2008) investigated actin–fimbrin/plastin interaction, and Chou et al. (Chou and Pollard, 2019) investigated ADP–actin binding. Cofilin (Tanaka et al., 2018), spectrin (Avery et al., 2017), and Arp2/3-binding sites (Zimmet et al., 2020) was also investigated using cryo-EM.

3D reconstruction techniques use Interactive Helical Real Space Reconstruction (IHRSR). The 3D reconstruction software using IHRSR has a heterogeneous structure compared to traditional Fourier–Bessel approaches. However, there is still limitation, because when the protein has large heterogeneity, a large proportion of densities become smeared out in the 3D structure after averaging. Therefore, researchers often use fixation to get tight ABP–actin binding or better alignment accuracy by checking the assigned azimuthal rotation angle of segments (Yang et al., 2016).

Structural studies of actin and ABPs using negative-stained EM and cryo-EM provide new insights into the mechanism underlying ABP–actin binding to regulate actin’s function. In addition, the structures show the competition between ABPs to regulate actin filaments’ interaction. Cofilin–actin and cofilin–actin bundles were also investigated using negative-stained EM (Minamide et al., 2010; Ngo et al., 2015). Hyperactive cofilin after dephosphorylation shows conformational changes forming rod-shaped cofilin- actin bundles, which can affect binding of other ABPs (Figure 3B; Ngo et al., 2015). The changes induce vesicle transport blocking, an increase in secreted Aβ, phosphorylated tau accumulation, neurofibrillary tangle (NFT) formation, and other pathological hallmarks of AD (Bamburg et al., 2010).

#### Electron Tomography Using Quick Freeze and Deep Etching

**FIGURE 4:**
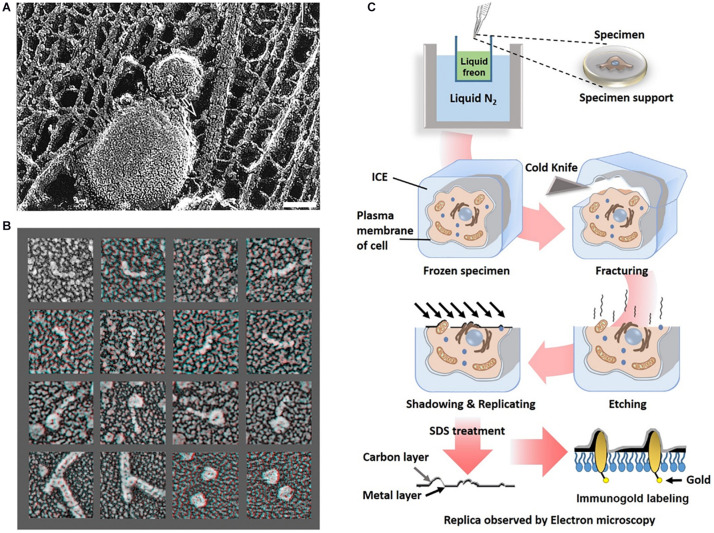
Freeze etching. **(A)** Organelle and cytoskeleton in mouse axon. A membranous organelle conveyed by transport is linked with a cytoskeleton, which could be a motor molecule (arrow). Scale bar = 50 nm. Reprinted by permission from Science (Hirokawa, 1998). **(B)** Deep etch micrograph of cortactin molecules (rows 1 and 2), Arp2/3 complexes with the ends of cortactin molecules (rows 3), F-actin “branchpoints” (Row 4: Left two panels), Arp2/3 molecules anone (Row 4: Right two panels). Reprinted by permission from Elsevier (Weaver et al., 2002). **(C)** A schematic diagram of freeze etching and freeze replica with immunolabeling.

Determining the organization of axonal actin filaments in neurons was challenging. Hirokawa used quick-freeze and deep-etch (QFDE) methods to prepare frog spinal nerve axons for EM (Hirokawa, 1982). While conventional transmission electron microscopy (cTEM) requires chemical fixation, dehydration, and plastic embedding and results in artifactual changes, QFDE EM provides a pseudo 3D appearance detailed structures. Freeze fracture involvers breaking a frozen sample to reveal its intercellular structures. Freeze etching is the sublimation of surface ice under vacuum to reveal originally hidden details of the fractured face (Figure 4C). With platinum replication using platinum, freeze-etched samples can be viewed with high dimensional stability and preservation quality capturing subtle structural changes, compared to cTEM (Hirokawa et al., 1988; Svitkina, 2009). QFDE EM can show a central region of microtubules, NFs, interconnected membranous organelles, and a dense actin filament and ABP network (Figure 4A). Therefore, QFDE EM has been used for decades to investigate cytoskeletal proteins (Weaver et al., 2002). Weaver et al. used deep-etch EM of cortactin, N-WASP (Neural Wiskott Aldrich Syndrome Protein), and the Arp2/3 complex and showed that cortactin and N-WASP can bind simultaneously to the Arp2/3 complex for actin assembly activation (Figure 4B). In addition, immunogold or fluorescent maker–labeled platinum-replica electron microscopy (PREM) used to study the specific ABPs. Labeling technique is a powerful tool to investigate the function of specific proteins (Hirokawa et al., 1988; Robenek and Severs, 2008; Vassilopoulos et al., 2019) in actin binding, and they will be each explained immuno EM and CLEM section.

**FIGURE 5:**
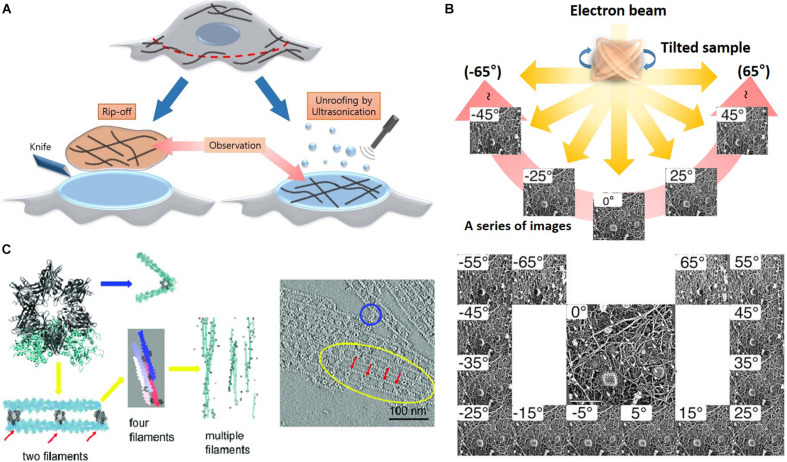
Advanced etching methods and electron tomography. **(A)** Rip off use adhesion unroofing method and ultrasonication is for mechanical unroofing. Reprinted by permission from Elsevier (Morone et al., 2008). **(B)** A high-resolution tomogram was obtained from serial tilt images. Schematic representation of electron tomography and each projection image was acquired at a different tilt angles. Reprinted by permission from Springer Nature (Morone, 2010). **(C)** Tomogram showing the spacing between adjacent CaMKII particles in the complexes is ∼36 nm. Reprinted by permission from PNAS (Wang Q. et al., 2019). Scale bar = 100 nm. ET, electron tomography.

Advanced methods (Figure 5) for visualizing cytoplasmic surface of the neuronal cell membrane (Morone et al., 2008) include mechanical shearing and adhesion unroofing. The mechanical shearing of a neuronal cell to expose the cytoplasmic surface of the neuronal cell membrane, and it enables observation of membrane cytoskeletons by cryo-EM and freeze-etch EM. In sonication unroofing, fine bubbles are generated, which adhere electrostatically to apical neuronal cell surfaces and remove the apical neuronal cell membrane (Figure 5A). In adhesion unroofing, positively charged grids that tightly adsorb neuronal cells are peeled from the cells. These techniques allowed visualization of the cytoplasmic surface and successfully shows many details of the cytoskeleton (Morone, 2010). In addition, it clearly shows the distribution of actin, ABPs, transmembrane proteins, membrane lipids, and some organelles. Advanced techniques using freeze-etch combined with electron tomography (ET) can visualize the 3D molecular architecture of membrane-associated structures (e.g., membrane skeleton, clathrin-coated pits, and caveolae) at high resolution (Morone, 2010). The nanometer resolution of the tomogram in Figure 5 shows that the spacing between adjacent proteins in the complexes is ∼36 nm. Freeze-etch electron tomography (ET) and ET using thin sectioning are good tools for high-resolution structural analysis. In ET, detailed 3D structures of subcellular macromolecular objects are obtained, because ET uses TEM images at incremental degrees of rotation around the target sample center. Images series of projections at different tilt angles (tilt series) (Figure 5B) are automatically aligned by cross-correlation or semiautomatic alignment with or without fiducial markers, such as gold particles. After the tilt-axis direction is determined by alignment, a tomogram is reconstructed using weighted back-projection (WBP), the algebraic reconstruction technique (ART), or the simultaneous iterative reconstruction technique (SIRT). Visualization of the computed tomogram is difficult because of neuronal cell complexity, so various segmentation techniques, such as artificial intelligence with manual segmentation, have been developed. A tomogram image showed actin filaments had in parallel or branching arrangements by ABPs, such as CaMKII (Figure 5C), in a neuron at a nanoscale resolution. ET showed improved resolution after sample preparation using quick freezing and cryo-EM (Figure 5C).

#### Immuno-Electron Microscopy

**FIGURE 6:**
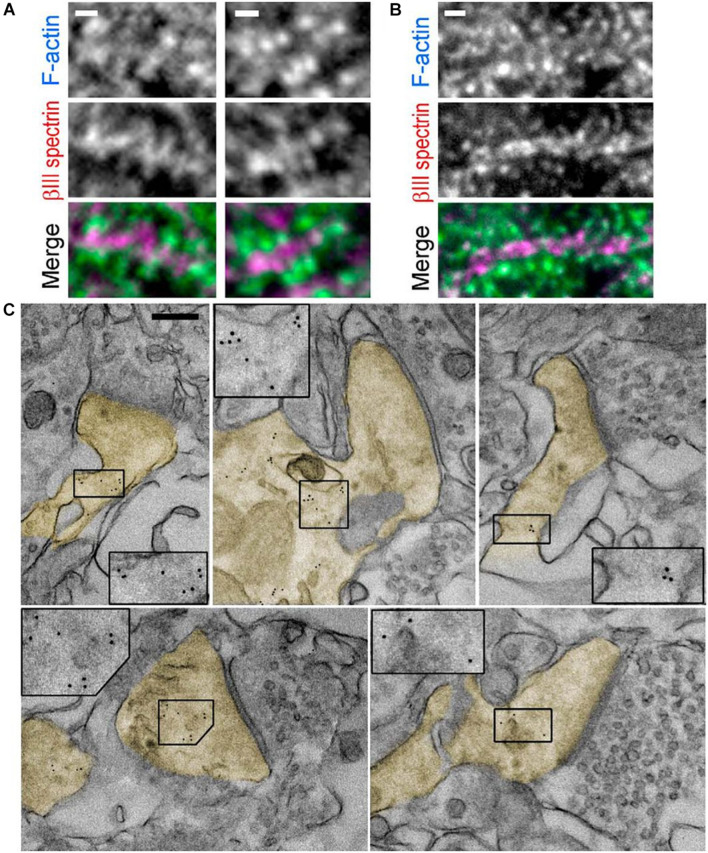
Immunogold labeling. **(A,B)**. Fluorescence staining with βIII spectrin antibody (magenta) and phalloidin (cyan) of tissue sections from mouse hippocampus. (C) Immunogold EM of βIII spectrin staining in thin sections of the mouse brain. Dendritic spines are pseudocolored in yellow. Inset: Boxed regions are enlarged to show 5 nm gold particles. Scale bars = **(A,B)**, 1 μm; **(C)**, 200 nm. EM, electron microscopy. Reprinted by permission from Society for Neuroscience (Efimova et al., 2017).

Immuno-EM is used to label proteins of interest using the antibody–antigen reaction like in indirect immunofluorescence. The first antibody of the antigen of interest and a second antibody-tagged colloidal gold particle are used to locate specific proteins for actin binding. Gold is used because of its high electron density, and the ABP location shows black dots in EM (Figure 6). Different sizes of gold particles can be used for multiple staining. Antibodies and gold particles cannot penetrate the resin used to embed samples, so before staining, thin sectioning using ultramicrotomy is necessary. For better antigenicity, cryofixation, freeze-substitution, low-temperature embedding, or cryo-sectioning is used in immuno-EM (Skepper and Powell, 2008). In addition, there are two different immunogold-labeling procedures, which can be divided depending on the embedding procedure. In pre-embedding, micrometer-thick tissue is immuno-stained prior to plastic embedding for ultrathin sections to be observed by TEM. In contrast, in post-embedding, tissue is embedded in a plastic resin and ultrathin sections are observed and then immunolabeled. Pre-embedding is better to get more immunolabeling, while post-embedding gives better ultrastructure (Webster et al., 2008). Immunogold labeling shows the detailed location of spectrin in the synapse (Figure 6C; Efimova et al., 2017) to study the function of βIII spectrin in neurons.

### Super-Resolution Fluorescence Microscopy

Super resolution microscopy can be divided into two categories; single-molecule localization microscopy (SMLM) and illumination pattern engineering methods. The SMLM use photo-switchable fluorophores to locate single molecules with high precision, and include stochastic optical reconstruction microscopy (STORM) (Rust et al., 2006), photo-activated localization microscopy (PALM) (Betzig et al., 2006), and fluorescence photoactivation localization microscopy (FPALM) (Hess et al., 2006). The illumination pattern engineering methods exploit a non-linear response to excitation fluorophores to overcome the diffraction limit. Such methods include stimulated emission depletion (STED) microscopy (Hell and Wichmann, 1994), [saturated] structured-illumination microscopy ([S]SIM) (Gustafsson et al., 1995), and reversible saturable optical fluorescence transitions (RESOLFT) microscopy (Hofmann et al., 2005). These SRM methods have also been applied to the study of actin and ABP by providing molecular-specific images at high resolution.

#### Stochastic Optical Reconstruction Microscopy (STORM)

**FIGURE 7:**
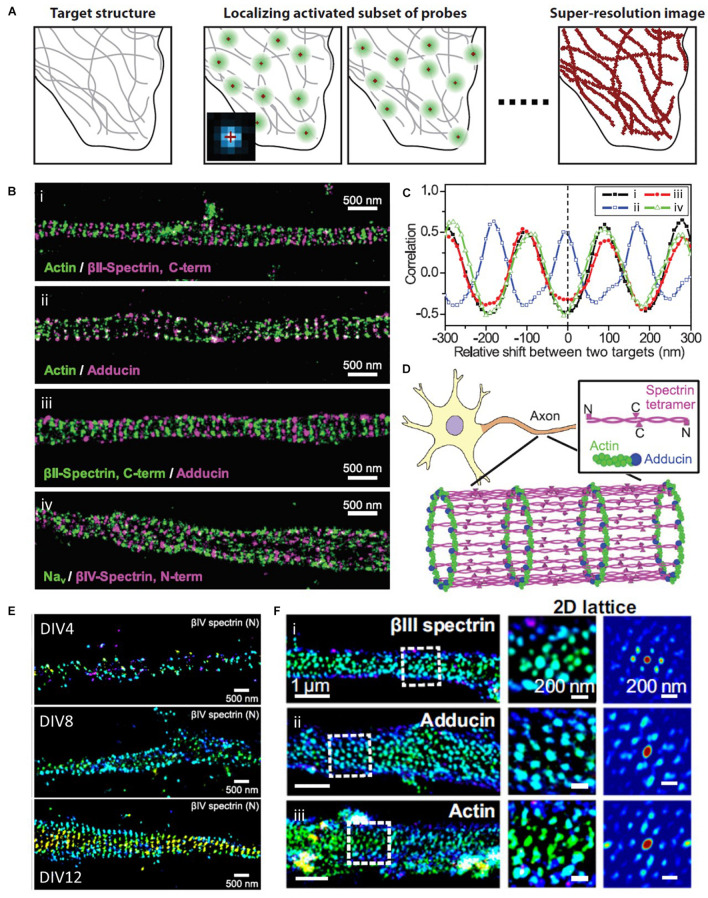
Super-resolution STORM images of actin and ABPs. **(A)** The principle of SMLM, such as STORM, PALM, and FPALM. Reprinted by permission from Annual Reviews (Huang et al., 2009). **(B)** Two-color STORM image of (i) actin (green) and βII-spectrin C terminus (magenta), (ii) actin (green) and adducin (magenta), (iii) βII-spectrin (green) and adducin (magenta), and (iv) sodium channels (Na_v_, green) and βIV-spectrin N terminus (magenta). **(C)** Spatial correlations between actin and the βII-spectrin C terminus [(i), black], between actin and adducin [(ii), blue], between adducin and the βII-spectrin C terminus [(iii), red], and between sodium channels and the βIV-spectrin N terminus [(iv), green], which are calculated for varying relative shifts between the two color channels along the axons. **(D)** A model for the cortical cytoskeleton in axons, forming ring-like structures wrapping around the circumference of the axon with a periodicity of ∼180 to 190 nm. Reprinted by permission from the American Association for the Advancement of Science **(B∼D)** (Xu et al., 2013). **(E)** Representative STORM images at different developmental stages, showing assembly of AIS components into the periodic lattice structure during late developmental stages. Reprinted by permission from Zhong et al. (2014). **(F)** MPS components form 2D polygonal lattice structures in some somatodendritic regions of neurons. (Left) 3D STORM image of a dendritic region from a DIV 28 mouse neuron stained for (i) βIII-spectrin, (ii) adducin, and (iii) βIII-spectrin. (Middle) Zoom-in image of the region indicated by the white dashed box in the Left. (Right) 2D autocorrelation function of this boxed region, which shows a 2D periodic lattice pattern. Reprinted by permission from PNAS **(E,F)** (Han et al., 2017).

The diffraction limit arises from the diffraction of light passed through optical systems, ultimately resulting in a blurred image from the spatial overlapping of point spread functions (PSF) of single molecules. To distinguish these molecules individually, STORM takes advantage of photo-switchable fluorescence dyes to temporally separate them (Figure 7A; Rust et al., 2006; Huang et al., 2009). The temporally separated individual molecules are localized with high precision, and a super-resolution image can be constructed from the collections of localizations from multiple fluorophores. This method enables the visualization of the ultrastructure of actin and ABP that was previously inaccessible with conventional optical methods and EM (Xu et al., 2013; Zhong et al., 2014; Sidenstein et al., 2016; Han et al., 2017; Pan et al., 2018; Wang G. et al., 2019). For example, Xu et al. (2013) first examined the periodic ring-like structure of actin, spectrin, adducin, and sodium channels in axons with a periodicity of ∼180–190 nm using STORM, which had not been resolved by conventional light microscopy (Figures 7B–D). They also performed two-color STORM imaging for spectrin and actin (or adducing), which showed alternating periodic structures. Such a quasi-1D, periodic, actin-spectrin cytoskeleton was not observed in dendrites; thus, it allowed the authors to speculate that this structure may play an essential role in providing stable and elastic mechanical support for axons, which need to withstand mechanical strains on their long and thin structures.

In a later study by the same research group, Zhong et al. (2014) used STORM to elucidate the developmental mechanism of this membrane-associated periodic skeleton (MPS), such as how periodic cytoskeletons form and why they primarily form in axons (Figure 7E). The STORM images revealed that this periodic structure is formed early on in the axon development, followed by propagation from the proximal to distal ends of axons. They also demonstrated that the local concentration of spectrin regulates this structure, and overexpression of spectrin induces this periodic structure in dendrites, which contain very low concentrations of spectrin under normal conditions. The authors also found that knocking out ankyrin B triggered the periodic structure in dendrites; thus, finding that ankyrin B is critical for the polarized distribution of βII spectrin in neurites.

Spectrin organization was also examined in a variety of neuronal and glial cell types by He et al. using the same methods (Sidenstein et al., 2016). The authors showed that spectrin has a long-range and periodic distribution throughout all axons, whereas small patches of periodic spectrin structures are present in the sub-regions of dendrites and in four types of glial cells. These findings allow the authors to conclude that the periodic organization of spectrin is conserved across a wide range of invertebrate and vertebrate animal species.

The spatial organization of spectrin, actin, and adducin in the dendrites and soma at different developmental stages of cultured hippocampal neurons was also investigated by STORM (Figure 7F; Han et al., 2017). Han et al. observed that one-dimensional MPS slowly develops in the dendrites of mature neurons at a much slower rate than that in axons. Interestingly, the authors also found that they form a two-dimensional polygonal lattice structure in the somatodendritic compartment at a much slower rate than that in the 1D MPS in axons. This result suggests that actin-based membrane skeletons organized with spectrin and ABP are differentially regulated across different sub-regions of neurons. A two-dimensional polygonal lattice structure was also observed from a native ultrastructure of the cytoskeleton in erythrocytes (Pan et al., 2018). Pan et al. used STORM imaging to resolve the ultrastructure of the cytoskeleton, including β-spectrin, F-actin, protein 4.1, tropomodulin, and adducin. They revealed a junction-to-junction distance of ∼80 nm, which is in agreement with the relaxed spectrin tetramers; actin and its capping proteins occupy subsets of junctional complexes.

Recently, Wang G. et al. (2019) investigated the behavior of MPS in sensory axon degeneration using the same super-resolution microscopy. From the STORM images of βII spectrin, they found that trophic deprivation (TD) caused the rapid disassembly of MPS, which can be initiated by actin destabilization. In contrast, knockout of βII spectrin prevented TD-induced retrograde signaling to protect axons from degeneration by inhibiting MPS formation.

Collectively, substantial progress has recently been made in identifying the developmental mechanism of the periodic actin-spectrin-based membrane skeleton in different sub-compartments of neurons by taking advantage of the high molecular specificity and spatial resolution of STORM.

#### Photo-Activated Localization Microscopy (PALM)

Another widely used SMLM method is PALM, which shares many similarities with STORM. PALM also exploits the temporal image separation of individual fluorophores based on the on/off behavior of fluorescent proteins (Betzig et al., 2006; Huang et al., 2009). Although the only difference between these two methods is the type of fluorescent label (i.e., photo-switchable fluorescent proteins for PALM and photo-switchable organic dyes for STORM), they are essentially similar in terms of configuration and differences are blurred as they use both fluorescent organic dyes and fluorescent proteins. PALM is also used in the study of actin, and most investigations have focused on the visualization of actin dynamics using single-molecule tracking (Tatavarty et al., 2009; Frost et al., 2010; Izeddin et al., 2011).

**FIGURE 8:**
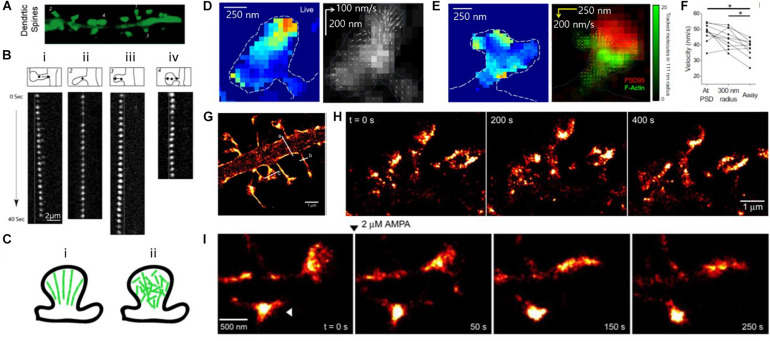
Super-resolution PALM images of actin, showing heterogeneous actin dynamics within individual spines. **(A)** Fluorescence images of mature dendritic spines (23DIV). **(B)** Time-lapse image sequences of single EosFP-actin molecules showing (i) retrograde, (ii) stationary, (iii) anterograde, and (iv) random direction movement. **(C)** Models of actin organization in dendritic spines. (left) Highly polarized actin cytoskeleton, which is inconsistent with the experimental results. (right) Weakly polarized actin cytoskeleton, which is more consistent with the experimental results. Reprinted by permission from Tatavarty et al. **(A∼C)** (Tatavarty et al., 2009). (**D**, Left) Map of actin molecule velocity across the inner extent of a dendritic spine, showing restricted areas of high velocity. (Right) Representative spine showing inward orientation of actin flow. (Arrow length: relative velocity) **(E)** (Left) Actin velocity map and (Right) locally averaged molecular movement vector on the deconvolved widefield image of PSD-95-cerulean (red) with local tracked molecule density (green), showing some but not all foci of high-velocity motion are closely associated with the synapse. **(D,E)** Reprinted by permission from Elsevier (Frost et al., 2010). **(F)** Average velocity of tracked particles, showing tracked molecules originating at or near the PSD moved faster than molecules originating elsewhere in the cell. **(D–F)** Reprinted by permission from Elsevier (Frost et al., 2010) **(G)** Super-resolution PALM image of the dendritic segment. **(H)** Super-resolution time-lapse imaging of dendritic spines from a neuron expressing ABP-tdEosFP at DIV 27, showing spine dynamics in mature hippocampal neurons. **(I)** PALM images of spine dynamics during AMPAR (2 mM) activation, showing rounding-up spine after treatment (arrowhead) Reprinted by permission from Izeddin et al. **(G∼I)** (Izeddin et al., 2011).

Tatavarty et al. (2009) for instance, utilized PALM to observe the kinematic (physical motion of actin filaments) and kinetic dynamics of F-actin in the dendritic spines of hippocampal neurons, which had been hindered by the small size of the dendritic spines in diffraction-limited conventional light microscopy (Figures 8A–C). Single-molecule tracking by PALM showed highly heterogeneous kinematic dynamics of F-actin in dendritic spines at a single-filament level, in which simultaneous actin movements were observed in both retrograde and anterograde directions. In contrast, the movements of filaments integrate into a net retrograde flow at the ensemble level, suggesting short actin filaments in dendritic spines.

Frost et al. (2010) also investigated the movement of individual actin molecules within living spines (Figures 8D–F). In a similar manner to Tatavarty et al., they used PALM-based single-molecule tracking to measure the velocity of single actin molecules along filaments as an index of the filament polymerization rate. They found that the actin velocity on filaments was substantially elevated at the synapses. In contrast, no enhanced polymerization activity was observed in the endocytic zone, implying a highly heterogeneous rate of actin molecules.

The morphological changes of the spine cytoskeleton were also investigated at super-resolution by Izeddin et al. (2011) (Figures 8G–I). By combining PALM imaging with quantum dot tracking for long-term imaging, they simultaneously observed the cytoskeleton and spine membranes, and their evolution during pharmacologically induced synaptic activity.

Although all studies discussed in this section visualize actin but not ABP in live cells, they demonstrate the possibility of future studies regarding ABP dynamics in relation to the actin in live neurons as a further application of PALM imaging.

#### Stimulated Emission Depletion (STED)

**FIGURE 9:**
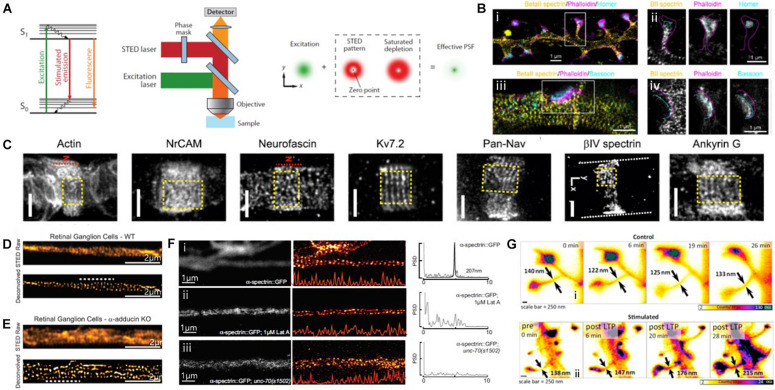
Super-resolution STED images of actin and ABPs. **(A)** The principle of STED microscopy. (Left) The stimulated emission process. (Middle) Schematic view of a STED microscope. (Right) A decreased size of the effective point spread function (PSF) by a donut-shaped STED laser. Reprinted by permission from Annual Reviews (Huang et al., 2009). **(B)** (i, iii) Three-color STED image of a dendrite decorated with spines and stained with βII spectrin, phalloidin, and Homer/Bassoon, showing the periodic spectrin organization. (ii, iv) Single-channel images of the spine indicated in (i), showing βII spectrin enters into the spine neck but does not reach the PSD. Magenta and cyan dashed lines highlight the shape of the spine/actin cage and the position of the PSD/Bassoon, respectively. Reprinted by permission from Springer Nature (Sidenstein et al., 2016) **(C)** Representative STED image of Glial actin, Axo-glial NrCAM, Neurofascin, Subunit 7.2 of axonal voltage-gated potassium channel (Kv7.2 or KCNQ2, N terminus antibody), axonal voltage-gated sodium channels (Pan-Na_v_), βIV spectrin, and Ankyrin G at nodal gaps of sciatic nerve fibers, some of which reveal a predominantly longitudinal periodicity. The red dashed line indicates the position of the node (“N”). Reprinted by permission from PNAS (D’este et al., 2017). **(D,E)** STED images of WT **(D)** and a-adducin KO **(E)** DIV19 RGCs, showing lack of adducin gives rise of axon actin rings of increased diameter in RGCs. Reprinted by permission from Leite et al. (2016). **(F)** Spectrin forms an actin-dependent, periodic cytoskeleton *in vitro*. Representative confocal (left), STED super-resolution images (center), and power spectral density (PSD) profiles (right) of a-spectrin SPC-1::GFP in C. elegans (i) control, (ii) control neurons treated with the actin depolymerizing agent, Latrunculin A (1 mM), (iii) in unc-70(s1502) b-spectrin null mutant axons *in vitro*. Reprinted by permission from Krieg et al. (2017) **(G)** STED images of dendritic spines observed under control conditions (i), as well as before and at subsequent times after chemical LTP stimulation (ii). Spine neck diameters are indicated in each frame by arrows. Reprinted by permission from Elsevier (Urban et al., 2011).

Another widely used SRM is STED, which was developed by Hell et al. In STED microscopy, a donut-shaped depletion light is used to suppress the fluorescence emission from the fluorophores as a pattern surrounding the focal spot of the excitation laser (Figure 9A; Hell and Wichmann, 1994; Huang et al., 2009). Such a negative pattern reduces the size of the fluorescent region and scanning of this sharpened focal spot generates a super-resolution image. Through the use of STED, actin and ABP have been thoroughly investigated (Urban et al., 2011; Lukinavičius et al., 2014; Leite et al., 2016; Sidenstein et al., 2016; Barabas et al., 2017; D’este et al., 2017; Krieg et al., 2017; Martínez et al., 2020).

For instance, the subcortical cytoskeleton organization at synaptic sites was previously investigated using three-color multilevel STED nanoscopy (Figure 9B; Sidenstein et al., 2016). This technique was developed based on an intrinsically co-aligned multicolor imaging scheme to overcome the limitations of multi-color imaging by STED, such as the requirement for complicated setups and analysis algorithms. This technique was successfully demonstrated by imaging neurofascin/spectrin and actin in cultured hippocampal neurons. The multicolor STED images revealed the ∼190 nm periodic actin/βII spectrin lattices along dendrites and spines, consistent with previously reported STORM images, whereas this periodic pattern was absent at presynaptic and postsynaptic sites. Such protein periodic structures in super-resolution images can now be automatically quantified in terms of the abundance and regularity of the MPS. For example, Barabas et al. (2017) presented a method for the automated quantification of the quality and abundance of periodic protein structures in super-resolution images as an open-source image analysis tool that provides the distributions of correlation coefficients for the spectrin membrane-associated periodic skeleton as an indication of the dynamic assembly and disassembly of the MPS.

D’este et al. (2017) also used STED to map the nanoscale molecular organization of 12 proteins at the nodes of Ranvier of sciatic nerve fibers, including cytoskeletal proteins of the axon, axonal, and glial nodal adhesion molecules, sodium and potassium channels, axonal proteins, and glial proteins (Figure 9C). The STED images showed that βIV spectrin and ankyrin G revealed a known one-dimensional longitudinal order at nodal gaps, whereas neurofascin-186 and neuron glial-related cell adhesion molecule (NrCAM) revealed a two-dimensional hexagonal-like lattice with a ∼190 nm periodicity, which has not yet been explored. The authors also observed that both glial proteins (neurofascin-155 and ankyrin B) and axonal proteins (βII spectrin, Caspr) form quasi-1D periodic arrangements at paranodes. These results suggest the importance of the lateral organization of proteins at the nodes of Ranvier.

The defects in ABP have also been investigated by STED imaging to better understand their roles. For example, Leite et al. (2016) observed an increased actin ring without changing the inter-ring periodicity in neurons in mice lacking adducing (Figures 9D,E). This result suggests a model in which adducin is responsible for controlling the diameter of actin rings and axons, but it is not necessary for actin ring assembly and periodicity. Krieg et al. (2017) reported that periodic spectrin structures were absent due to the loss of UNC-70 β-spectrin and actin depolymerization from STED images (Figure 9F). These results allowed the authors to conclude that mechanical neuroprotection depends on spectrin-dependent longitudinal tension, and the defects in β-spectrin may sensitize neurons to damage.

Live cell imaging for actin was also performed using time-lapse STED imaging. To investigate synapses in brain slices or brains under realistic conditions, Urban et al. (2011) used aberration-reducing optics for STED imaging and demonstrated this for deep inside biological tissue imaging (Figure 9G). This method enabled the visualization of distinct actin distributions inside dendritic spines in living organotypic brain slices at depths of up to 120 mm with 60–80 nm spatial resolution, revealing that structurally heterogeneous and highly dynamic actin in spine necks were modulated by neuronal activity. However, long-term STED imaging of live biological samples is often limited due to its high laser power requirement for high spatial resolution. To overcome this limit, Lukinavičius et al. (2014) introduced fluorogenic probes for actin and tubulin (SiR-actin, SiR-tubulin) that exhibited minimal cytotoxicity, excellent brightness, and photostability. They successfully revealed the one-dimensional periodic actin organization in the axons and the nine-fold symmetry of the centrosome of cultured rat neurons. These advances are expected to allow further investigations into ABP dynamics *in vivo* in the near future.

### Correlative Light and Electron Microscopy (CLEM)

As previously stated, fluorescence light microscopy (LM) and EM have been extensively used for biological studies to probe cellular structures and obtain a better understanding of biological functions. Each of these techniques has its distinct strengths and weaknesses that complement each other, as previously discussed. Fluorescence LM provides protein-specific information with high molecular specificity and sensitivity. Multicolor fluorescence LM offers a promising approach to monitor potential molecular interactions between spectrally different fluorescent labels, and live cell LM imaging can reveal the spatiotemporal dynamics of a protein. Moreover, recently developed SRM techniques allow images to be taken with a higher resolution than that with the diffraction limit, as described in the previous section.

Although such advances in SRM have made remarkable headway in improving spatial resolution by order of magnitude or moreover, the diffraction limit, EM generally achieves a higher spatial resolution in protein localization compared to that with SRM, revealing the subcellular anatomy at resolutions of nanometers. However, conventional fixation and staining methods of traditional EM do not usually provide protein-specific information because of the limited molecular specificity. Moreover, EM is limited to live cell imaging, since the electrons destroy the samples, and the sample needs to be processed and fixed for EM imaging.

As the strengths and weaknesses of LM and EM are complementary, these two methods can be effective if used together for the same sample. This combination is the so-called correlative light and EM (CLEM). It provides different and complementary information by merging the specific protein localization advantages of LM with the ultrastructural information of EM.

Correlative light and electron microscopy is often compared to immuno-EM because this has been the method of choice for protein localization at an ultrastructural level. However, the application of immuno-EM is significantly limited by several technical difficulties, including the destruction of antigens by strong fixation, inaccessibility of antigens in the plastic, poor survival of epitopes, limited preservation of morphology, incomplete labeling, non-specific binding of antibodies, and non-specific background. Therefore, only a subset of target molecules are successfully detected by immuno-EM. These drawbacks have prompted the development of various CLEMs for protein localization at an ultrastructural level. Compared with immuno-EM, CLEM significantly improves the detection efficiency and sensitivity with sufficient reliability and specificity. Moreover, when EM is combined with SRM, such correlative SRM-EM approaches provide a more meaningful correlation between two images due to their closer resolution match compared to that by CLEM (Kim et al., 2015).

**FIGURE 10:**
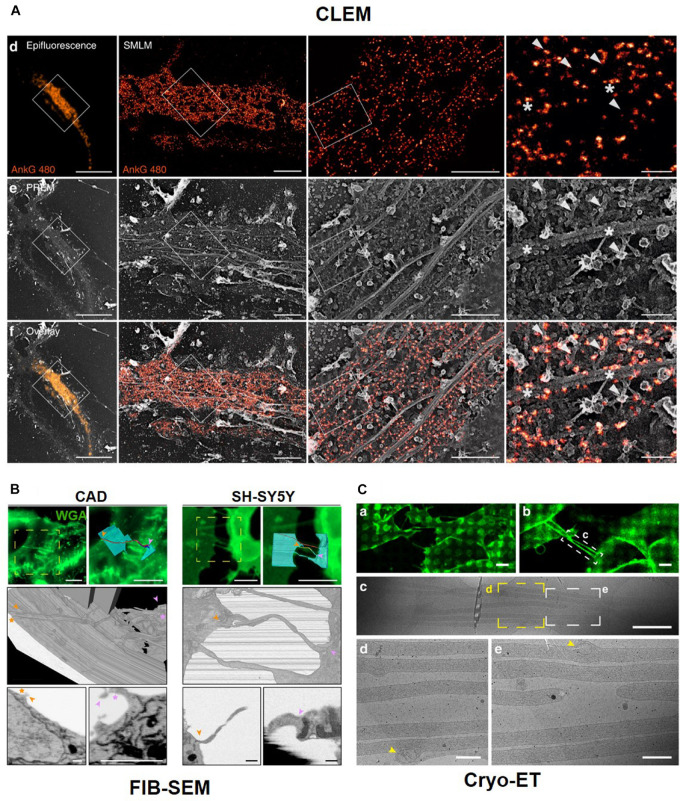
Correlative Light and Electron Microscopy. **(A)** Correlative results of Ankyrin G tail (ankG 480, orange) proximal axon by fluorescent microscope image and PREM image. Reprinted by permission from Springer Nature (Vassilopoulos et al., 2019). CLEM with Serial block-face (SBF)-SEM technique **(B)** and Cryo-Electron tomography **(C)**. Reprinted by permission from Springer Nature (Sartori-Rupp et al., 2019).

Such CLEM methods have also allowed for the identification of neuronal cell structures and processes of interest in whole-cell images with high molecular specificity and high spatial resolution. This dual examination provides valuable unique and complementary information for actin and ABP. For instance, Vassilopoulos et al. (2019) utilized PREM to visualize the dynamic actin network and combined this with STORM to specifically localize ankyrin G (Figure 10A). This correlation imaging method revealed the molecular organization of the actin rings and MPS components along the axonal plasma membrane. In particular, the association of MPS with microtubules via ankyrin G was visualized along the AIS by correlative STORM and PREM.

Sochacki et al. also showed the technique of CLEM with PREM to observe a clathrin lattice and actin (Sochacki et al., 2017). CLEM is also was used to obtain high-resolution images using serial sectioning. For example, tunneling nanotubes (TNTs), which are actin-rich membranous protrusions, play a role in the intercellular transport of various cargoes between neuronal cells and can be observed by CLEM, which reveals their structural identity using LM with focused ion beam (FIB)–scanning electron microscopy (SEM) in neuronal cells (Figure 10B). FIB-SEM is a serial block-face (SBF)-SEM technique using serial sectioning. Another SBF-SEM technique uses an ultramicrotome embedded inside a SEM. The advantage of these techniques is that we can easily obtain serial ultrathin sections, but the sections can be observed at one time. CLEM can be combined with cryo-ET after cryo-FIB sectioning. CLEM can also be combined with confocal or fluorescent microscopy and super-resolution microscopy, such as stochastic optical reconstruction microscopy (STORM) (Zhanghao et al., 2019) and stimulated emission depletion microscopy (STED) (Urban et al., 2011). Cryo-EM and cryo-FIB-SEM with LM show open-ended TNTs labeled with anti-*N*-cadherin antibodies and 3D structures of TNTs. Labeled TNTs are filled with parallel actin bundles and membrane-bound mitochondria (Figure 10C; Sartori-Rupp et al., 2019).

## Conclusion and Future Perspectives

In conclusion, various microscopy techniques have been successfully demonstrated to visualize actin filaments and ABPs in neuronal cells. First of all, EM has been the primary tool to study actin and ABPs because of its nanometer resolution, for example, single particle analysis, electron tomography with freeze etching and unroof, and immuno-gold staining. Using these EM techniques, ABP binding sites for actin filaments and the organization of axonal actin filaments in neurons have been investigated. However, molecular specific imaging and live-cell imaging have been limited to EM techniques. To overcome these limitations, the recently developed SRM has been applied to the study of actin and ABP, including STORM, PALM, and PALM. This method enables the visualization of the ultrastructure of actin and ABP that was previously inaccessible with conventional optical methods and EM, for example, the periodic ring-like structure of actin and ABPs even in live neurons with high molecular specificity at high resolution. As the strengths and weaknesses of LM and EM are complementary, the CLEM has also been applied to the study of actin and ABP. Combining these two methods has allowed for the identification of neuronal cell structures and processes of interest in whole cells with high molecular specificity and high spatial resolution.

In the future, we foresee that *in situ* or wet-environment EM would be a goal in this field to study actin and ABPs. Studies have reported progress in observing proteins’ structural changes (Fitzpatrick et al., 2013) and the ultrastructure of living cells in the Four-dimension (4D), time (De Jonge and Ross, 2011; Ross, 2015). Although the resolution is poor, live cells or proteins *in situ* have been observed so that actin and ABPs would be the next challenge for this 4D EM-combined CLEM in the near future.

## Author Contributions

MJ, DK, and JM wrote the manuscript. All authors contributed to the article and approved the submitted version.

## Conflict of Interest

The authors declare that the research was conducted in the absence of any commercial or financial relationships that could be construed as a potential conflict of interest.

## References

[B1] Avery A. W. Fealey M. E. Wang F. Orlova A. Thompson A. R. Thomas D. D. (2017). Structural basis for high-affinity actin binding revealed by a beta-III-spectrin SCA5 missense mutation. *Nat. Commun.* 8:1350. 10.1038/s41467-017-01367-w 29116080 PMC5676748

[B2] Bamburg J. R. Bernstein B. W. Davis R. C. Flynn K. C. Goldsbury C. Jensen J. R. (2010). ADF/Cofilin-actin rods in neurodegenerative diseases. *Curr. Alzheimer Res.* 7 241–250. 10.2174/156720510791050902 20088812 PMC4458070

[B3] Barabas F. M. Masullo L. A. Bordenave M. D. Giusti S. A. Unsain N. Refojo D. (2017). Automated quantification of protein periodic nanostructures in fluorescence nanoscopy images: abundance and regularity of neuronal spectrin membrane-associated skeleton. *Sci. Rep.* 7 1–10. 10.1038/s41598-017-16280-x 29167561 PMC5700202

[B4] Bartesaghi A. Merk A. Banerjee S. Matthies D. Wu X. Milne J. L. S. (2015). 2.2 Å resolution cryo-EM structure of β-galactosidase in complex with a cell-permeant inhibitor. *Science* 348 1147–1151. 10.1126/science.aab1576 25953817 PMC6512338

[B5] Behrmann E. Tao G. Stokes D. L. Egelman E. H. Raunser S. Penczek P. A. (2012). Real-space processing of helical filaments in SPARX. *J. Struct. Biol.* 177 302–313. 10.1016/j.jsb.2011.12.020 22248449 PMC3288516

[B6] Betzig E. Patterson G. H. Sougrat R. Lindwasser O. W. Olenych S. Bonifacino J. S. (2006). Imaging intracellular fluorescent proteins at nanometer resolution. *Science* 313 1642–1645. 10.1126/science.1127344 16902090

[B7] Borovac J. Bosch M. Okamoto K. (2018). Regulation of actin dynamics during structural plasticity of dendritic spines: signaling messengers and actin-binding proteins. *Mol. Cell Neurosci.* 91 122–130. 10.1016/j.mcn.2018.07.001 30004015

[B8] Cho Y. Park D. Cavalli V. (2015). Filamin A is required in injured axons for HDAC5 activity and axon regeneration. *J. Biol. Chem.* 290 22759–22770. 10.1074/jbc.m115.638445 26157139 PMC4566247

[B9] Chou S. Z. Pollard T. D. (2019). Mechanism of actin polymerization revealed by cryo-EM structures of actin filaments with three different bound nucleotides. *Proc. Natl. Acad. Sci. U.S.A.* 116 4265–4274. 10.1073/pnas.1807028115 30760599 PMC6410863

[B10] Cichon J. Sun C. Chen B. Jiang M. Chen X. A. Sun Y. (2012). Cofilin aggregation blocks intracellular trafficking and induces synaptic loss in hippocampal neurons. *J. Biol. Chem.* 287 3919–3929. 10.1074/jbc.m111.301911 22184127 PMC3281697

[B11] Craig R. (2017). Molecular structure of muscle filaments determined by electron microscopy. *Appl. Micros.* 47 226–232. 10.9729/am.2017.47.4.226 29682492 PMC5909410

[B12] Das S. Ge P. Oztug Durer Z. A. Grintsevich E. E. Zhou Z. H. Reisler E. (2020). D-loop dynamics and near-atomic-resolution Cryo-EM structure of phalloidin-bound F-Actin. *Structure* 28:e583. 10.1016/j.str.2020.04.004 32348747 PMC7316398

[B13] De Jonge N. Ross F. M. (2011). Electron microscopy of specimens in liquid. *Nat. Nanotechnol.* 6 695–704. 10.1038/nnano.2011.161 22020120

[B14] D’este E. Kamin D. Balzarotti F. Hell S. W. (2017). Ultrastructural anatomy of nodes of Ranvier in the peripheral nervous system as revealed by STED microscopy. *Proc. Natl. Acad. Sci. U.S.A.* 114 E191–E199. 10.1073/pnas.1619553114 28003466 PMC5240729

[B15] Dogterom M. Koenderink G. H. (2019). Actin–microtubule crosstalk in cell biology. *Nat. Rev. Mol. Cell Biol.* 20 38–54. 10.1038/s41580-018-0067-1 30323238

[B16] Efimova N. Korobova F. Stankewich M. C. Moberly A. H. Stolz D. B. Wang J. (2017). betaIII spectrin is necessary for formation of the constricted neck of dendritic spines and regulation of synaptic activity in neurons. *J. Neurosci.* 37 6442–6459. 10.1523/jneurosci.3520-16.2017 28576936 PMC5511878

[B17] Egelman E. H. (2010). Reconstruction of helical filaments and tubes. *Methods Enzymol.* 482 167–183. 10.1016/s0076-6879(10)82006-3 20888961 PMC3245864

[B18] Fitzpatrick A. W. Lorenz U. J. Vanacore G. M. Zewail A. H. (2013). 4D cryo-electron microscopy of proteins. *J. Am. Chem. Soc.* 135 19123–19126. 10.1021/ja4115055 24313395

[B19] Fromm S. A. Sachse C. (2016). Cryo-EM structure determination using segmented helical image reconstruction. *Methods Enzymol.* 579 307–328. 10.1016/bs.mie.2016.05.034 27572732

[B20] Frost N. A. Shroff H. Kong H. Betzig E. Blanpied T. A. (2010). Single-molecule discrimination of discrete perisynaptic and distributed sites of actin filament assembly within dendritic spines. *Neuron* 67 86–99. 10.1016/j.neuron.2010.05.026 20624594 PMC2904347

[B21] Galkin V. E. Orlova A. Cherepanova O. Lebart M. C. Egelman E. H. (2008). High-resolution cryo-EM structure of the F-actin-fimbrin/plastin ABD2 complex. *Proc. Natl. Acad. Sci. U.S.A.* 105 1494–1498. 10.1073/pnas.0708667105 18234857 PMC2234172

[B22] Galkin V. E. Orlova A. Kudryashov D. S. Solodukhin A. Reisler E. Schroder G. F. (2011). Remodeling of actin filaments by ADF/cofilin proteins. *Proc. Natl. Acad. Sci. U.S.A.* 108 20568–20572. 10.1073/pnas.1110109108 22158895 PMC3251117

[B23] Gray K. T. Kostyukova A. S. Fath T. (2017). Actin regulation by tropomodulin and tropomyosin in neuronal morphogenesis and function. *Mol. Cell Neurosci.* 84 48–57. 10.1016/j.mcn.2017.04.002 28433463 PMC5648638

[B24] Griffiths G. Hoppeler H. (1986). Quantitation in immunocytochemistry: correlation of immunogold labeling to absolute number of membrane antigens. *J. Histochem. Cytochem.* 34 1389–1398. 10.1177/34.11.3534077 3534077

[B25] Grintsevich E. E. Galkin V. E. Orlova A. Ytterberg A. J. Mikati M. M. Kudryashov D. S. (2010). Mapping of drebrin binding site on F-actin. *J. Mol. Biol.* 398 542–554. 10.1016/j.jmb.2010.03.039 20347847 PMC2866048

[B26] Gustafsson M. G. Agard D. A. Sedat J. W. (1995). “Sevenfold improvement of axial resolution in 3D wide-field microscopy using two objective-lenses,” in *Proceedings of the Three-Dimensional Microscopy: Image Acquisition and Processing II*: International Society for Optics and Photonics, (Washington, DC: SPIE - International Society for Optics and Photonics), 147–156. 10.1117/12.205334

[B27] Han B. Zhou R. Xia C. Zhuang X. (2017). Structural organization of the actin-spectrin–based membrane skeleton in dendrites and soma of neurons. *Proc. Natl. Acad. Sci. U.S.A.* 114 E6678–E6685. 10.1073/pnas.1705043114 28739933 PMC5559029

[B28] Hell S. W. Wichmann J. (1994). Breaking the diffraction resolution limit by stimulated emission: stimulated-emission-depletion fluorescence microscopy. *Optics Lett.* 19 780–782. 10.1364/ol.19.000780 19844443

[B29] Hess S. T. Girirajan T. P. Mason M. D. (2006). Ultra-high resolution imaging by fluorescence photoactivation localization microscopy. *Biophys. J.* 91 4258–4272. 10.1529/biophysj.106.091116 16980368 PMC1635685

[B30] Hirokawa N. (1982). Cross-linker system between neurofilaments, microtubules, and membranous organelles in frog axons revealed by the quick-freeze, deep-etching method. *J. Cell Biol.* 94 129–142. 10.1083/jcb.94.1.129 6181077 PMC2112203

[B31] Hirokawa N. (1998). Kinesin and dynein superfamily proteins and the mechanism of organelle transport. *Science* 279 519–526. 10.1126/science.279.5350.519 9438838

[B32] Hirokawa N. Hisanaga S. Shiomura Y. (1988). MAP2 is a component of crossbridges between microtubules and neurofilaments in the neuronal cytoskeleton: quick-freeze, deep-etch immunoelectron microscopy and reconstitution studies. *J. Neurosci.* 8 2769–2779. 10.1523/jneurosci.08-08-02769.1988 3045269 PMC6569399

[B33] Hofmann M. Eggeling C. Jakobs S. Hell S. W. (2005). Breaking the diffraction barrier in fluorescence microscopy at low light intensities by using reversibly photoswitchable proteins. *Proc. Natl. Acad. Sci. U.S.A.* 102 17565–17569. 10.1073/pnas.0506010102 16314572 PMC1308899

[B34] Hotulainen P. Llano O. Smirnov S. Tanhuanpaa K. Faix J. Rivera C. (2009). Defining mechanisms of actin polymerization and depolymerization during dendritic spine morphogenesis. *J. Cell Biol.* 185 323–339. 10.1083/jcb.200809046 19380880 PMC2700375

[B35] Huang B. Bates M. Zhuang X. (2009). Super-resolution fluorescence microscopy. *Annu. Rev. Biochem.* 78 993–1016. 10.1146/annurev.biochem.77.061906.092014 19489737 PMC2835776

[B36] Izeddin I. Specht C. G. Lelek M. Darzacq X. Triller A. Zimmer C. (2011). Super-resolution dynamic imaging of dendritic spines using a low-affinity photoconvertible actin probe. *PLoS One* 6:e15611. 10.1371/journal.pone.0015611 21264214 PMC3022016

[B37] Kevenaar J. T. Hoogenraad C. C. (2015). The axonal cytoskeleton: from organization to function. *Front. Mol. Neurosci.* 8:44. 10.3389/fnmol.2015.00044 26321907 PMC4536388

[B38] Kim D. Deerinck T. J. Sigal Y. M. Babcock H. P. Ellisman M. H. Zhuang X. (2015). Correlative stochastic optical reconstruction microscopy and electron microscopy. *PLoS One* 10:e0124581. 10.1371/journal.pone.0124581 25874453 PMC4398493

[B39] Krieg M. Stühmer J. Cueva J. G. Fetter R. Spilker K. Cremers D. (2017). Genetic defects in β-spectrin and tau sensitize C. *elegans* axons to movement-induced damage via torque-tension coupling. *eLife* 6:e20172. 10.7554/eLife.20172 28098556 PMC5298879

[B40] Kuhn S. Geyer M. (2014). Formins as effector proteins of Rho GTPases. *Small GTPases* 5:e29513. 10.4161/sgtp.29513 24914801 PMC4111664

[B41] Leite S. C. Sampaio P. Sousa V. F. Nogueira-Rodrigues J. Pinto-Costa R. Peters L. L. (2016). The actin-binding protein α-adducin is required for maintaining axon diameter. *Cell Rep.* 15 490–498. 10.1016/j.celrep.2016.03.047 27068466 PMC4838511

[B42] Lukinavičius G. Reymond L. D’este E. Masharina A. Göttfert F. Ta H. (2014). Fluorogenic probes for live-cell imaging of the cytoskeleton. *Nat. Methods* 11 731–733. 10.1038/nmeth.2972 24859753

[B43] Ma Q. L. Yang F. Frautschy S. A. Cole G. M. (2012). PAK in Alzheimer disease. Huntington disease and X-linked mental retardation. *Cell Logist.* 2 117–125. 10.4161/cl.21602 23162743 PMC3490962

[B44] Marsick B. M. San Miguel-Ruiz J. E. Letourneau P. C. (2012). Activation of ezrin/radixin/moesin mediates attractive growth cone guidance through regulation of growth cone actin and adhesion receptors. *J. Neurosci.* 32 282–296. 10.1523/jneurosci.4794-11.2012 22219290 PMC3306234

[B45] Martínez G. F. Gazal N. G. Quassollo G. Szalai A. M. Del Cid-Pellitero E. Durcan T. M. (2020). Quantitative expansion microscopy for the characterization of the spectrin periodic skeleton of axons using fluorescence microscopy. *Sci. Rep.* 10 1–11. 10.1038/s41598-020-59856-w 32076054 PMC7031372

[B46] Matusek T. Gombos R. Szecsenyi A. Sanchez-Soriano N. Czibula A. Pataki C. (2008). Formin proteins of the DAAM subfamily play a role during axon growth. *J. Neurosci.* 28 13310–13319. 10.1523/jneurosci.2727-08.2008 19052223 PMC6671601

[B47] Merk A. Fukumura T. Zhu X. Darling J. E. Grisshammer R. Ognjenovic J. (2020). 1.8 A resolution structure of beta-galactosidase with a 200 kV CRYO ARM electron microscope. *IUCr J.* 7 639–643. 10.1107/s2052252520006855 32695410 PMC7340270

[B48] Minamide L. S. Maiti S. Boyle J. A. Davis R. C. Coppinger J. A. Bao Y. (2010). Isolation and characterization of cytoplasmic cofilin-actin rods. *J. Biol. Chem.* 285 5450–5460. 10.1074/jbc.m109.063768 20022956 PMC2820773

[B49] Morone N. (2010). Freeze-etch electron tomography for the plasma membrane interface. *Methods Mol. Biol.* 657 275–286. 10.1007/978-1-60761-783-9_22 20602224

[B50] Morone N. Nakada C. Umemura Y. Usukura J. Kusumi A. (2008). Three-dimensional molecular architecture of the plasma-membrane-associated cytoskeleton as reconstructed by freeze-etch electron tomography. *Methods Cell Biol.* 88 207–236. 10.1016/s0091-679x(08)00412-3 18617036

[B51] Narita A. Maeda Y. (2007). Molecular determination by electron microscopy of the actin filament end structure. *J. Mol. Biol.* 365 480–501. 10.1016/j.jmb.2006.06.056 17059832

[B52] Ngo K. X. Kodera N. Katayama E. Ando T. Uyeda T. Q. (2015). Cofilin-induced unidirectional cooperative conformational changes in actin filaments revealed by high-speed atomic force microscopy. *eLife* 4:e04806 10.7554/eLife.04806.033 PMC4337605 25642645

[B53] Noguchi J. Hayama T. Watanabe S. Ucar H. Yagishita S. Takahashi N. (2016). State-dependent diffusion of actin-depolymerizing factor/cofilin underlies the enlargement and shrinkage of dendritic spines. *Sci. Rep.* 6:32897. 10.1038/srep32897 27595610 PMC5011767

[B54] Pan L. Yan R. Li W. Xu K. (2018). Super-resolution microscopy reveals the native ultrastructure of the erythrocyte cytoskeleton. *Cell Rep.* 22 1151–1158. 10.1016/j.celrep.2017.12.107 29386104

[B55] Pant K. Chereau D. Hatch V. Dominguez R. Lehman W. (2006). Cortactin binding to F-actin revealed by electron microscopy and 3D reconstruction. *J. Mol. Biol.* 359 840–847. 10.1016/j.jmb.2006.03.065 16697006

[B56] Pinyol R. Haeckel A. Ritter A. Qualmann B. Kessels M. M. (2007). Regulation of N-WASP and the Arp2/3 complex by Abp1 controls neuronal morphology. *PLoS One* 2:e400. 10.1371/journal.pone.0000400 17476322 PMC1852583

[B57] Robenek H. Severs N. J. (2008). Recent advances in freeze-fracture electron microscopy: the replica immunolabeling technique. *Biol Proced Online* 10 9–19. 10.1251/bpo138 18385807 PMC2275045

[B58] Robinson J. Takizawa T. Vandre D. (2000). Applications of gold cluster compounds in immunocytochemistry and correlative microscopy: comparison with colloidal gold. *J. Microscopy* 199 163–179. 10.1046/j.1365-2818.2000.00734.x 10971797

[B59] Ross F. M. (2015). Opportunities and challenges in liquid cell electron microscopy. *Science* 350:aaa9886. 10.1126/science.aaa9886 26680204

[B60] Rust M. J. Bates M. Zhuang X. (2006). Sub-diffraction-limit imaging by stochastic optical reconstruction microscopy (STORM). *Nat. Methods* 3 793–796. 10.1038/nmeth929 16896339 PMC2700296

[B61] Sarmiere P. D. Bamburg J. R. (2004). Regulation of the neuronal actin cytoskeleton by ADF/cofilin. *J Neurobiol.* 58 103–117. 10.1002/neu.10267 14598374

[B62] Sartori-Rupp A. Cordero Cervantes D. Pepe A. Gousset K. Delage E. Corroyer-Dulmont S. (2019). Correlative cryo-electron microscopy reveals the structure of TNTs in neuronal cells. *Nat. Commun.* 10:342. 10.1038/s41467-018-08178-7 30664666 PMC6341166

[B63] Sidenstein S. C. D’este E. Böhm M. J. Danzl J. G. Belov V. N. Hell S. W. (2016). Multicolour multilevel STED nanoscopy of Actin/Spectrin Organization at Synapses. *Sci. Rep.* 6:26725. 10.1038/srep26725 27220554 PMC4879624

[B64] Skepper J. N. Powell J. M. (2008). Immunogold staining following freeze substitution and low temperature embedding after chemical fixation or after cryoimmobilization for transmission electron microscopy (TEM). *CSH Protoc.* 2008:dbrot5017. 10.1101/pdb.prot5017 21356851

[B65] Sochacki K. A. Dickey A. M. Strub M. P. Taraska J. W. (2017). Endocytic proteins are partitioned at the edge of the clathrin lattice in mammalian cells. *Nat. Cell Biol.* 19 352–361. 10.1038/ncb3498 28346440 PMC7509982

[B66] Soria Fregozo C. Perez Vega M. I. (2012). Actin-binding proteins and signalling pathways associated with the formation and maintenance of dendritic spines. *Neurologia* 27 421–431. 10.1016/j.nrleng.2011.10.006 22178050

[B67] Strasser G. A. Rahim N. A. Vanderwaal K. E. Gertler F. B. Lanier L. M. (2004). Arp2/3 is a negative regulator of growth cone translocation. *Neuron* 43 81–94. 10.1016/j.neuron.2004.05.015 15233919

[B68] Svitkina T. (2009). Imaging cytoskeleton components by electron microscopy. *Methods Mol. Biol.* 586 187–206. 10.1007/978-1-60761-376-3_10 19768431 PMC2925411

[B69] Tanaka K. Takeda S. Mitsuoka K. Oda T. Kimura-Sakiyama C. Maeda Y. (2018). Structural basis for cofilin binding and actin filament disassembly. *Nat. Commun.* 9:1860. 10.1038/s41467-018-04290-w 29749375 PMC5945598

[B70] Tatavarty V. Kim E. J. Rodionov V. Yu J. (2009). Investigating sub-spine actin dynamics in rat hippocampal neurons with super-resolution optical imaging. *PLoS One* 4:e7724. 10.1371/journal.pone.0007724 19898630 PMC2771285

[B71] Thompson P. M. Tolbert C. E. Shen K. Kota P. Palmer S. M. Plevock K. M. (2014). Identification of an actin binding surface on vinculin that mediates mechanical cell and focal adhesion properties. *Structure* 22 697–706. 10.1016/j.str.2014.03.002 24685146 PMC4039106

[B72] Trinick J. Cooper J. Seymour J. Egelman E. H. (1986). Cryo-electron microscopy and three-dimensional reconstruction of actin filaments. *J Microsc* 141 349–360. 10.1371/journal.pone.0007724 3701854

[B73] Unsain N. Stefani F. D. Caceres A. (2018). The Actin/Spectrin membrane-associated periodic skeleton in neurons. *Front. Synaptic. Neurosci.* 10:10. 10.3389/fnsyn.2018.00010 29875650 PMC5974029

[B74] Urban N. T. Willig K. I. Hell S. W. Nägerl U. V. (2011). STED nanoscopy of actin dynamics in synapses deep inside living brain slices. *Biophys. J.* 101 1277–1284. 10.1016/j.bpj.2011.07.027 21889466 PMC3164186

[B75] Vassilopoulos S. Gibaud S. Jimenez A. Caillol G. Leterrier C. (2019). Ultrastructure of the axonal periodic scaffold reveals a braid-like organization of actin rings. *Nat. Commun.* 10:5803. 10.1038/s41467-019-13835-6 31862971 PMC6925202

[B76] Von Der Ecken J. Muller M. Lehman W. Manstein D. J. Penczek P. A. Raunser S. (2015). Structure of the F-actin-tropomyosin complex. *Nature* 519 114–117. 10.1038/nature14033 25470062 PMC4477711

[B77] Wang G. Simon D. J. Wu Z. Belsky D. M. Heller E. O’rourke M. K. (2019). Structural plasticity of actin-spectrin membrane skeleton and functional role of actin and spectrin in axon degeneration. *eLife* 8:e38730 10.7554/eLife.38730.041 PMC6494423 31042147

[B78] Wang Q. Chen M. Schafer N. P. Bueno C. Song S. S. Hudmon A. (2019). Assemblies of calcium/calmodulin-dependent kinase II with actin and their dynamic regulation by calmodulin in dendritic spines. *Proc. Natl. Acad. Sci. U.S.A.* 116 18937–18942. 10.1073/pnas.1911452116 31455737 PMC6754556

[B79] Weaver A. M. Heuser J. E. Karginov A. V. Lee W.-L. Parsons J. T. Cooper J. A. (2002). Interaction of Cortactin and N-WASp with Arp2/3 Complex. *Curr. Biol.* 12 1270–1278. 10.1016/s0960-9822(02)01035-7 12176354

[B80] Webster P. Schwarz H. Griffiths G. (2008). Preparation of cells and tissues for immuno EM. *Methods Cell Biol.* 88 45–58. 10.1016/s0091-679x(08)00403-2 18617027

[B81] Winkelman J. D. Bilancia C. G. Peifer M. Kovar D. R. (2014). Ena/VASP Enabled is a highly processive actin polymerase tailored to self-assemble parallel-bundled F-actin networks with Fascin. *Proc. Natl. Acad. Sci. U.S.A.* 111 4121–4126. 10.1073/pnas.1322093111 24591594 PMC3964058

[B82] Xu K. Zhong G. Zhuang X. (2013). Actin, spectrin, and associated proteins form a periodic cytoskeletal structure in axons. *Science* 339 452–456. 10.1126/science.1232251 23239625 PMC3815867

[B83] Yang S. Woodhead J. L. Zhao F. Q. Sulbaran G. Craig R. (2016). An approach to improve the resolution of helical filaments with a large axial rise and flexible subunits. *J. Struct. Biol.* 193 45–54. 10.1016/j.jsb.2015.11.007 26592473 PMC4696882

[B84] Zhanghao K. Chen X. Liu W. Li M. Liu Y. Wang Y. (2019). Super-resolution imaging of fluorescent dipoles via polarized structured illumination microscopy. *Nat. Commun.* 10:4694. 10.1038/s41467-019-12681-w 31619676 PMC6795901

[B85] Zhong G. He J. Zhou R. Lorenzo D. Babcock H. P. Bennett V. (2014). Developmental mechanism of the periodic membrane skeleton in axons. *eLife* 3:e04581 10.7554/eLife.04581.025 PMC4337613 25535840

[B86] Zimmet A. Van Eeuwen T. Boczkowska M. Rebowski G. Murakami K. Dominguez R. (2020). Cryo-EM structure of NPF-bound human Arp2/3 complex and activation mechanism. *Sci. Adv.* 6:eaaz7651. 10.1126/sciadv.aaz7651 32917641 PMC7274804

